# Evidence for action: a call for the global HIV response to address the needs of transgender populations

**DOI:** 10.7448/IAS.19.3.21193

**Published:** 2016-07-17

**Authors:** Tonia C Poteat, JoAnne Keatley, Rose Wilcher, Chloe Schwenke

**Affiliations:** 1 Department of Epidemiology, Johns Hopkins School of Public Health, Baltimore, MD, USA; 2 Center of Excellence for Transgender Health, University of California, San Francisco, CA, USA; 3 Research Utilization Department, FHI 360, Durham, NC, USA; 4 McCourt School of Public Policy, Georgetown University, Washington, DC, USA

Transgender people are severely underserved in the global response to HIV. Less than 40% of countries report that their national AIDS strategies address transgender people [1], despite a growing body of evidence that transgender women, in particular, face a disproportionate and heavy burden of HIV. An estimated 19% of transgender women worldwide are living with HIV, and they have almost 50 times the odds of living with HIV compared to other reproductive age adults [2]. The impact among transgender sex workers is even more profound. Transgender women sex workers have a prevalence of HIV that is nine times that of non-transgender female sex workers and three times that of male sex workers [3].

Data on HIV among transgender men are extremely limited. However, emerging studies among transgender men who have sex with men (MSM) suggest heightened HIV vulnerability among this group. While specific data on transgender men are lacking, in settings with high HIV prevalence and epidemics of gender-based violence, sexual assault on gender variant persons places transgender men and women at substantial risk for HIV as well as other negative sequelae of sexual violence. The stigma, violence and human rights abuses transgender people suffer drive much of their risk for HIV and hinder their access to care.

While the world's response to HIV has largely overlooked transgender people and the myriad factors that increase their risk, the tide is slowly turning. In 2014, the World Health Organization provided guidance on the essential elements of HIV programming among key populations, including the first specific recommendations for transgender people [4]. More recently, leaders in transgender health have spearheaded the development and launch of the first practical guide for implementing HIV and STI programmes with transgender people [5]. These documents represent important steps forward in the global HIV response. However, in order to provide the most effective interventions to the populations with the greatest need, we need research that is specific to the unique concerns of transgender communities. This calls for research to be accessible to all who seek to implement transgender-competent, evidence-based programmes. This special issue of the *Journal of the International AIDS Society* is dedicated to that goal.

We issued a global call for abstracts addressing topics relevant to HIV in transgender populations, and submissions by transgender authors were encouraged. The response was overwhelming. More than 80 abstracts were submitted! After a careful process of editorial and peer review, we selected 11 high-quality manuscripts that contribute data and document experiences from a variety of geographic regions and social contexts. This supplement brings to the forefront new empiric data, case studies and commentaries that, taken together, expand our understanding of the HIV epidemic in transgender communities and offer practical, rights-based and evidence-informed recommendations for reducing HIV and promoting the broader health and human rights of transgender individuals.

## Epidemiological research

We include three original research articles that present new epidemiological and behavioural data addressing HIV risk and prevalence among transgender people in countries and regions with little published research. The supplement opens with a study by Stahlman and colleagues characterizing the HIV-related risks of transgender women as compared with MSM in three West African countries – Togo, Burkina Faso and Cote d'Ivoire [6]. The data from this study's sample of 453 transgender women found that transgender women were more likely than MSM to report high-risk behaviours, including engaging in condomless anal intercourse and sex work. In addition, transgender women were more likely to be living with HIV as compared with MSM (19% vs. 7%). Experiencing social stigma was significantly associated with condomless anal intercourse and sex work among the study population.

Kaplan *et al*. offer insights on the HIV-related risks and needs of transgender women in the Middle East and North Africa region [7]. This study with 53 transgender women from Lebanon found an HIV prevalence of 10% among those who self-reported being HIV-positive or participated in testing. However, a quarter of participants declined to be tested, suggesting the HIV prevalence in this population may be higher. The study found participants to be at high risk of acquiring and transmitting HIV with 57% of participants having had condomless receptive anal intercourse in the past three months and two-thirds currently engaging in sex work. All but one participant reported experiencing transgender identity-related discrimination, and 68% had experienced violence because of their gender identity. Participation in sex work and being open about transgender identity were associated with condomless receptive anal intercourse.

Cai *et al*.'s study with 220 transgender women sex workers in China also contributes data on HIV prevalence and condomless receptive anal intercourse with male partners [8]. More than a quarter (25.9%) of the study sample was HIV-positive. Similar to the study from Lebanon, however, the authors acknowledge this is likely an underestimate given that 40% of the participants declined to be tested as part of the study. Nearly 27% of all participants and one-third of those who were identified as HIV-positive reported engaging in condomless receptive anal intercourse in the past month. Efforts to avoid revealing transgender identity to male clients were associated with condomless receptive anal intercourse while condom self-efficacy was associated with lower likelihood of condomless intercourse.

## New evidence to advance community- and clinic-based practice

Two manuscripts expand the evidence base for how programme implementers and service delivery providers can more effectively support transgender people to be healthy and empowered. Shaikh and colleagues report outcomes of the Pechan programme in India supported by the Global Fund, contributing critical programmatic evidence from one of the only large-scale, trans-specific programmes to be evaluated to date [9]. The evaluation found that the programme's rights-based, community-led empowerment and gender-affirming approach produced several significant outcomes among transgender individuals in states across India, including increased use of tailored HIV, health, legal, social and psychological services; increased condom use; increased self-efficacy; as well as collective efficacy and identity.

A manuscript by Radix *et al*. highlights the need for more data to guide clinical care recommendations for transgender women living with HIV who are accessing both gender-affirming hormone therapy and antiretroviral therapy (ART) [10]. The authors describe the feminizing hormonal regimens used by many transgender women and summarize the available data on drug–drug interactions between feminizing hormonal agents and ART. Most of the data investigating oestrogens and ART came from studies of oral contraceptives; no studies were found that examined interactions between ART and the types and doses of oestrogens found in feminizing regimens. The authors call for more research in this area and encourage providers to closely monitor patients for drug interactions and potential side-effects that may lead to loss of virologic suppression.

## Data on the programmatic needs of key transgender sub-populations

While available data suggest that HIV prevalence is the greatest among adult transgender women, we were interested in evidence addressing the needs of various sub-groups within the transgender population. To that end, we include two manuscripts specific to transgender men and transgender youth.

Scheim and colleagues draw on data from a global survey of MSM to assess access to HIV prevention services for a sub-sample of 69 transgender MSM [11]. The study found that access to services among transgender MSM was inadequate with only 43.5, 53.6 and 26.1% of respondents having access to HIV testing, condoms and lubricants, respectively. Moreover, transgender MSM were less likely to have access to HIV testing and lubricants compared with non-transgender MSM. The study found that ever having been arrested or convicted due to being transgender and having experienced stigma from a healthcare provider posed significant barriers to accessing prevention services.

A study by Le *et al*. with 301 transgender female youth in the United States contributed data on the relationships between parental acceptance, sources of social support, mental health and HIV risk factors [12]. Data from this study suggest that transgender female youth whose parents are their primary source of social support experience greater parental acceptance of their transgender identity and less psychological distress than those who have non-parental primary social support. However, only 16.3% of the respondents reported having parental primary social support. No significant associations were found between type of primary social support and HIV risk factors such as age at sexual debut and engagement in condomless anal sex. Nevertheless, the authors conclude that interventions focused on parental acceptance of their child's gender identity may improve young transgender people's access to parental social support, which could mitigate risks of HIV and poor mental health outcomes over the life course.

## Field-based policy and programmatic case studies

The supplement contains two manuscripts documenting progress and gaps in addressing the HIV-related needs of transgender communities in the Latin America region. Silva-Santisteban and colleagues provide an assessment of the state of HIV prevention programmes for transgender women in 17 countries in Latin America, examining the quality, scope and effectiveness of programme implementation; accessibility and coverage; existing legal frameworks; levels of transgender community participation; and how well each country's programmatic approach aligns with international recommendations [13]. The authors describe how Argentina, Brazil, Mexico and Uruguay have prevention strategies that are specifically tailored for transgender women, with Argentina and Uruguay singled out as having a human rights–based approach that emphasizes social inclusion. However, other countries failed to demonstrate trans-specific programmes. The authors note that, while there are some promising new interventions, the existing limited coverage of services, entrenched stigma and discrimination and the pervasive distrust of the healthcare system among transgender women constitute important obstacles to effective HIV prevention in the region.

A more encouraging picture emerges in a case study by Salazar *et al*., which describes how a remarkable collaboration involving multiple stakeholders resulted in the development of a robust national strategy for addressing HIV among transgender women in Peru [14]. The policy is responsive to the leading determinants of the HIV epidemic among transgender women in that country, including socio-economic conditions, the living and working environment, accessibility of health services, the existence of social networks, as well as other pertinent lifestyle and biological factors. Appropriate consideration was also given to identifying effective strategies to ameliorate specific legal and human rights challenges, stigma and violence experienced by the Peruvian transgender population. The authors attribute a decade-long process of evidence generation, policy dialogue and capacity building with the transgender community as key factors leading to this significant policy success.

## Calls to action

We close the supplement with two commentaries that provide specific calls to action to address overarching issues experienced by transgender people globally. Divan and colleagues juxtapose the range of international and regional human rights protections with the lived experience of transgender people who suffer the consequences of punitive and discriminatory laws that deny and threaten their very existence [15]. The authors make an urgent call for countries to address the human rights violations of transgender people in order to honour international obligations, stem HIV epidemics, promote gender equality, strengthen social and economic development and put a stop to untrammelled violence.

The final commentary by Wolf and colleagues draws attention to recently developed programmatic tools with the potential to meaningfully advance the health and human rights of transgender populations, if widely implemented [16]. These tools – supported by multi-national organizations such as the Pan American Health Organization, World Health Organization, U.S. President's Emergency Plan for AIDS Relief and the United Nations Development Programme, in collaboration with regional transgender groups – provide concrete recommendations for interventions that respond to the health, HIV, violence, stigma and discrimination, social protection, and human rights needs of transgender communities. The authors urge a range of audiences, from government ministers to programme implementers, to immediately put these tools to use for trans-specific advocacy, training, strategic planning, capacity building and programme design.

## Where we go from here

In addition to their contributions to the literature on HIV among transgender populations, each of the manuscripts in this supplement underscores the specific vulnerabilities and unique needs of transgender populations. It cannot be overstated that transgender women are not MSM and should not be subsumed in a category that erases their identity. Likewise, transgender MSM should be visible within the data about MSM. The contents of this supplement also make clear that addressing stigma, discrimination and structural violence is essential for any effective HIV response for transgender communities. Now is the time for funders to invest more in research and programmes tailored for transgender communities and for implementers and providers to design and deliver trans-competent programmes and services. On the whole, we hope that the manuscripts in this supplement not only confirm the urgency of HIV epidemics in transgender communities around the world but also provide readers with compelling case studies and global resources they can use to create an effective response, led by and grounded in the human rights of transgender people.
